# Real-Time Detection Algorithm for Kiwifruit Canker Based on a Lightweight and Efficient Generative Adversarial Network

**DOI:** 10.3390/plants12173053

**Published:** 2023-08-25

**Authors:** Ying Xiang, Jia Yao, Yiyu Yang, Kaikai Yao, Cuiping Wu, Xiaobin Yue, Zhenghao Li, Miaomiao Ma, Jie Zhang, Guoshu Gong

**Affiliations:** 1 College of Information Engineering, Sichuan Agricultural University, Ya’an 625000, China; 202005513@stu.sicau.edu.cn (Y.X.); 201902257@stu.sicau.edu.cn (J.Y.); 202005781@stu.sicau.edu.cn (Y.Y.); 202106029@stu.sicau.edu.cn (X.Y.); 202106047@stu.sicau.edu.cn (Z.L.); 2 Sichuan Key Laboratory of Agricultural Information Engineering, Ya’an 625000, China; 3 College of Agronomy, Sichuan Agricultural University, Chengdu 611130, China; yaokaikai@stu.sicau.edu.cn (K.Y.); 71262@sicau.edu.cn (C.W.); mmma@sicau.edu.cn (M.M.)

**Keywords:** crop protection, deep learning, computer vision, kiwifruit bacterial canker, few-shot processing, generative adversarial network, disease detection, smart agriculture

## Abstract

Disease diagnosis and control play important roles in agriculture and crop protection. Traditional methods of identifying plant disease rely primarily on human vision and manual inspection, which are subjective, have low accuracy, and make it difficult to estimate the situation in real time. At present, an intelligent detection technology based on computer vision is becoming an increasingly important tool used to monitor and control crop disease. However, the use of this technology often requires the collection of a substantial amount of specialized data in advance. Due to the seasonality and uncertainty of many crop pathogeneses, as well as some rare diseases or rare species, such data requirements are difficult to meet, leading to difficulties in achieving high levels of detection accuracy. Here, we use kiwifruit trunk bacterial canker (*Pseudomonas syringae* pv. *actinidiae*) as an example and propose a high-precision detection method to address the issue mentioned above. We introduce a lightweight and efficient image generative model capable of generating realistic and diverse images of kiwifruit trunk disease and expanding the original dataset. We also utilize the YOLOv8 model to perform disease detection; this model demonstrates real-time detection capability, taking only 0.01 s per image. The specific contributions of this study are as follows: (1) a depth-wise separable convolution is utilized to replace part of ordinary convolutions and introduce noise to improve the diversity of the generated images; (2) we propose the GASLE module by embedding a GAM, adjust the importance of different channels, and reduce the loss of spatial information; (3) we use an AdaMod optimizer to increase the convergence of the network; and (4) we select a real-time YOLOv8 model to perform effect verification. The results of this experiment show that the Fréchet Inception Distance (FID) of the proposed generative model reaches 84.18, having a decrease of 41.23 compared to FastGAN and a decrease of 2.1 compared to ProjectedGAN. The mean Average Precision (mAP@0.5) on the YOLOv8 network reaches 87.17%, which is nearly 17% higher than that of the original algorithm. These results substantiate the effectiveness of our generative model, providing a robust strategy for image generation and disease detection in plant kingdoms.

## 1. Introduction

Globally, kiwifruit is a highly valued fruit species due to its unique taste and nutritional benefits, making it a popular choice among consumers [1]. However, the large-scale cultivation of kiwifruit has led to the perennial prevalence of kiwifruit bacterial canker, which is caused by *Pseudomonas syringae* pv. *actinidiae*. These pathogenic bacteria can invade the xylem of the kiwifruit trunk and cause local ulcerative decay, affecting the transportation and absorption of nutrients within the trunk and ultimately leading to severe tree mortality, which poses a serious threat to the kiwi industry [2]. Traditionally, the diagnosis of this disease in kiwifruit has primarily relied on visual assessments conducted by experts in the identification and judgment of plant diseases. However, this method lacks real-time monitoring capabilities, making it challenging to promptly detect and respond to disease outbreaks. At the same time, it is difficult for agricultural producers to accurately quantify and assess the extent of disease damage based on the actual condition of the crop.

The early diagnosis of plant diseases via computer vision technology offers a crucial advantage in effectively controlling the large-scale spread of plant diseases and eliminating them at their initial stages, as it not only improves crop yield and quality, but also significantly reduces the use of pesticides. The study of kiwifruit disease detection has important implications for the agricultural field, helping growers eliminate the disease in its early stages, as well as safeguarding the growers’ economic rights with respect to cultivation while ensuring the food safety of consumers.

Recently, with the continuous development of artificial intelligence technology, both domestic and foreign researchers have begun to apply advanced image processing and computer vision technologies to the fields of plants and agriculture. For example, Wang et al. [3] used the YOLOV5s algorithm based on channel pruning to achieve rapid detection of apple fruits, thus realizing yield estimation at an early stage. In addition, Sozzi et al. [4] applied several versions of the YOLO algorithm to automate the detection of grape bunches of white grape varieties, thus evaluating the accuracy and speed of different detection techniques in practice. Similarly, Cardellicchio et al. [5] applied the YOLOV5 algorithm to detect the phenotypic features of tomato plants, enabling the monitoring of the tomato growth process and yield prediction. However, it is worth noting that object detection technology based on computer vision is also gradually being implemented in plant disease feature extraction [6]. In 2021, Wang et al. [7] conducted a study of rice disease identification based on multi-model transfer learning. In 2022, Yao et al. [8] proposed a two-stage algorithm to identify kiwifruit leaf diseases in natural settings, effectively distinguishing between various diseases affecting kiwifruit leaves. In 2022, Lin et al. [9] proposed a lightweight CNN model to identify the various symptoms and stages of a specific grape disease. Using object detection technology to detect plant diseases not only saves time and energy, but also enables real-time judgment, allowing prompt intervention and control measures to be taken. By quickly identifying and diagnosing diseases, timely actions can be implemented to mitigate their spread and minimize associated losses in crop yield and quality. This approach holds great research value and significance, as it helps to develop effective agricultural disease management strategies.

In the plant domain, computer vision tasks often require a substantial quantity of annotated images to train models. However, obtaining a substantial quantity of annotated image data in the plant domain is often challenging and time consuming. Since different plants have different growth stages and performance traits, the distribution of data in existing plant datasets may be unbalanced, and there may be less coverage of rare plant species or diseases than common species and diseases. Additionally, plant distributions can vary regionally and seasonally, making it difficult to create image datasets that encompass these diversities. Correspondingly, in cases of datasets that cannot encompass these diversities of plants, the generation ability of a model may be limited, making it difficult to achieve accurate identification in different environments [10].

Currently, the approach most commonly employed to address data insufficiency is image augmentation, which expands the dataset by applying geometric transformations or introducing color variations. However, this strategy can introduce a certain level of noise and inaccuracy, which may negatively impact the performance of the model. Another approach that has gained popularity is the utilization of pre-trained models on large-scale image datasets in the form of transfer learning. Utilizing the knowledge acquired from these pre-existing models, researchers aim to expedite the training process and enhance the performance of their experiments. However, the pre-trained models may possess features and concepts that do not match the specific plant domain under study, resulting in relatively poor model performance.

Recently, generative adversarial networks (GANs) have made significant progress in the field of image generation, which has been driven by deep learning techniques [11,12,13]. Many advanced GANs, such as BigGAN [14], have demonstrated impressive results by leveraging large-scale datasets for training. This study will apply a lightweight GAN to deal with the problem of insufficient data being available for plant disease detection.

This paper provides a new idea to solve the above problem. Taking kiwifruit trunk bacterial canker as an example, we first collected a high-quality few-sample dataset of bacterial canker in kiwifruit tree trunk samples. Based on this dataset, we used a lightweight image generation model with the generator of FastGAN [15] and the discriminator of ProjectedGAN [16] as the backbone for data expansion. In order to further improve the quality of the generated images and, thus, make them more helpful when performing the detection task, we investigated the architecture of the generator in greater depth. We utilized a depth-wise separable convolution (DSC) [17,18] to build deeper generator structures and avoid incurring additional computational overheads. At the same time, we introduced the GASLE module to enable the generator to focus on more efficient information and prevent the problem of localized collapse. In addition, in order to increase the stability of training and accelerate convergence, we employed the AdaMod [19] optimizer. Finally, we used the advanced YOLOV8 model to detect disease sites.

## 2. Results

### 2.1. Image Generation

**Figure 1:**
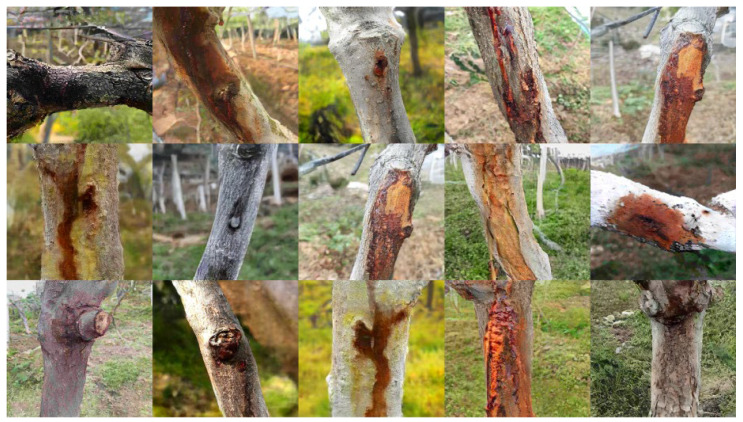
An example of our generated images.

Compared to the original FastGAN and ProjectedGAN, our generative model has obvious advantages in terms of generating images of kiwi tree trunks. The improved generative model demonstrates a minimal reduction in the computational speed while achieving an enhancement in the Fréchet inception distance (FID). The optimal FIDs of each model are illustrated in Table 1. Figure 1 shows our generated images.

Our proposed model demonstrates a substantial improvement in the FID, with a reduction of 41.23 compared to FastGAN and a reduction of 2.1 compared to ProjectedGAN. Compared to previous models, our model generates images that are closer to real images in terms of their visual quality and diversity. Figure 1 depicts some generated images.

**Figure 2:**
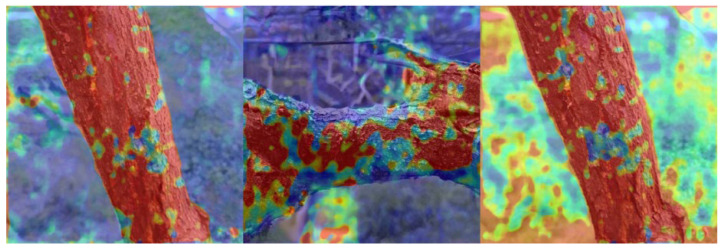
Visualization of attention weights during generation.

In order to enhance our comprehension of the learning process within our generative model, we visualized the attention weights associated with different images. Figure 2 shows some examples. It can be seen that our network can better focus on the key features in the images, allowing more precise control over the generation process, thereby generating more realistic images.

### 2.2. Object Detection

**Figure 3:**
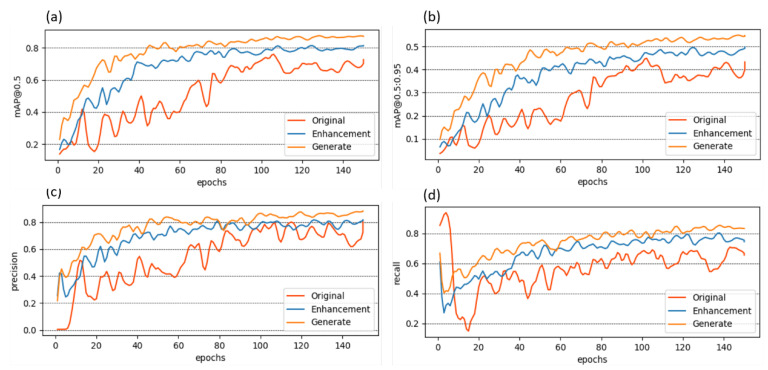
YOLOv8 results: (**a**) mAP@0.5; (**b**) mAP@0.5:0.95; (**c**) precision; (**d**) recall.

To analyze the effectiveness of our proposed method in detecting kiwifruit trunk disease and compare it to existing data augmentation techniques, we created multiple datasets to train the YOLOv8 network. These datasets included the original dataset, the image augmentation dataset, and the image generation dataset. We allocated the training data and validation data in an 8:2 distribution and trained them for 150 epochs. The experimental results are presented in Figure 3.

Due to the scarcity of samples, complex image backgrounds, and imbalanced data distribution in the original dataset, using it to train an object detection model results in instability and a tendency toward premature convergence. After multiple training epochs, we selected the best-performing model, as shown by the original curve, which achieved a mAP@0.5 of 70.93%. By applying the appropriate image augmentation techniques, the mAP@0.5 based on YOLOv8 improved to 75.94%. After further incorporating the images generated via our proposed model, the mAP@0.5 reached 87.17%. Notably, the improvements in precision and recall were also significant.

The experimental results demonstrate that our proposed method substantially enhances the accuracy of kiwifruit bacterial canker detection. Furthermore, the authenticity of the generated images and their applicability to real scenes are convincingly demonstrated. These findings provide strong evidence to support the effectiveness and utility of our method to address the challenge of accurately detecting and diagnosing kiwifruit bacterial canker.

### 2.3. Ablation Experiment

To enhance the generator’s performance, we introduced three modules: DSC, GASLE, and AdaMod. To ascertain the efficacy of our model, we conducted ablation experiments, and the corresponding results are illustrated in Table 2. The specific experimental method was used to embed these three modules into the generator and generate corresponding datasets. We used YOLOv8 to perform training and detection, and the object detection results are illustrated in the figures below.

**Figure 4:**
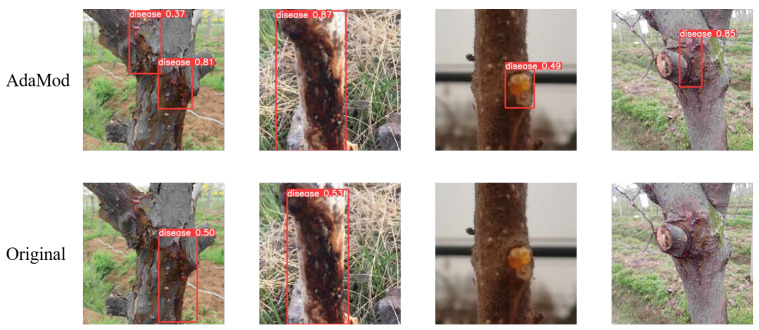
Detection examples from AdaMod and the original dataset.

As can be seen from the above table, the three modules all help the model to generate higher-quality and more reliable images. Among these modules, the DSC module is the most obvious. In order to further analyze the effectiveness of the model, we conducted a more detailed analysis of the generated data. Firstly, the detection samples for the AdaMod module are illustrated in Figure 4:

In the figure above, it can be seen that the data generated after the introduction of AdaMod are more realistic and more readily detectable via YOLOv8. At the same time, the level of detection confidence is higher for the AdaMod images, and the judgment of the model is more accurate.

**Figure 5:**
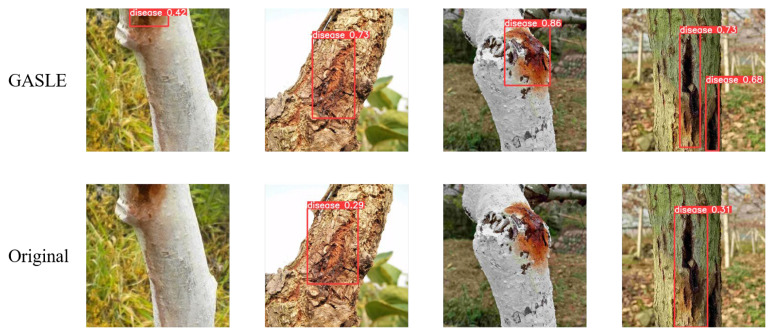
Detection examples for GASLE and the original dataset.

Secondly, there is the GASLE module, for which detection samples are illustrated in Figure 5.

In the figure shown above, it can be observed that the data generated after the introduction of GASLE are more realistic and can be detected more easily via YOLOv8. At the same time, GASLE pays more attention to important features, making YOLOv8’s judgment more accurate, reducing the misjudgment of non-disease areas, and increasing the level of detection confidence.

**Figure 6:**
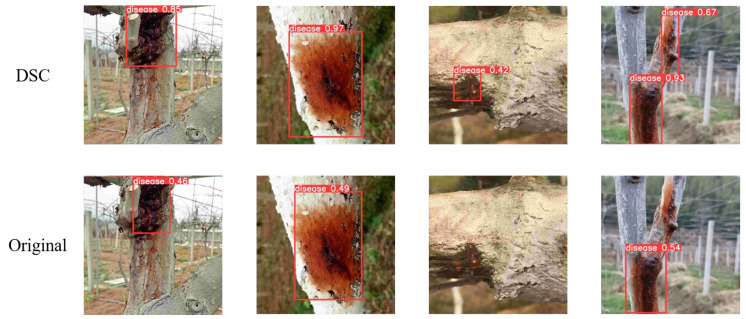
Detection examples for DSC and the original dataset.

The last module tested is the DSC module, the detection samples for which are illustrated in Figure 6.

In the above figure, it can be observed that the quality of the images generated after the introduction of DSC is improved, the overall authenticity and generation of local features are more realistic, the disease is easier to detect via YOLOv8, and the level of detection confidence is higher.

## 3. Materials and Methods

### 3.1. Generative Adversarial Networks

**Figure 7:**
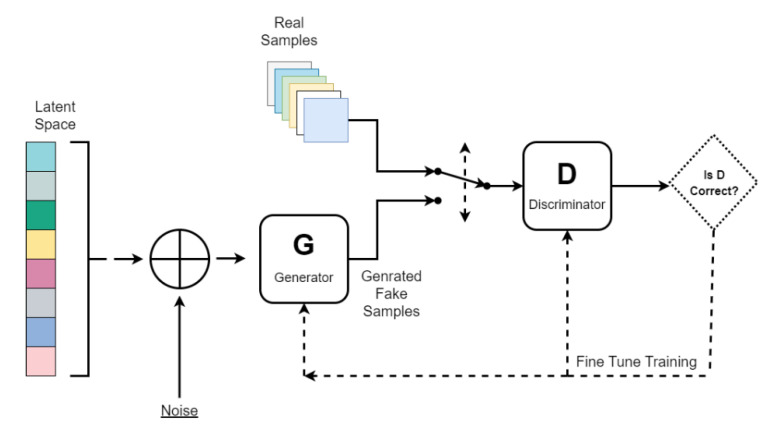
Generative adversarial network.

GANs were first proposed in 2014 by Goolow et al. [20] and have since become some of the models most commonly utilized in the domain of unconditional image generation. The basic structure of a GAN consists of two fundamental components: a generator (G) and a discriminator (D). The G’s role is to produce new data instances similar to the input images, while the D’s role is to evaluate the authenticity of the generated images and separate them from the real images. These two components are jointly trained via a min–max game in which the G strives to generate images that can deceive the D, while the D strives to distinguish between real and fake data [20]. During training, the G learns to generate increasingly realistic images to deceive the D. At the same time, the D learns to improve its ability to discriminate between the fake images generated via the G and the real images. This process is illustrated in Figure 7.

### 3.2. Data Acquisition

The images used in this paper were captured at the Ya’an kiwifruit plantation in Dujiangyan, Sichuan Province, China. A Canon EOS60D DSLR camera with a resolution of 1920 × 1080 pixels was utilized to capture the dataset. After removing the redundant background via data processing techniques such as center cropping, we obtained a high-quality 520 × 520-pixel kiwifruit trunk disease dataset. The initial few-shot dataset consisted of 128 images, which laid the foundation for the subsequent experiments and analysis.

### 3.3. The Generator

#### 3.3.1. The Structure of the Generator

In general, the design of the generator’s structure is typically deepened to synthesize higher-resolution and more realistic images. However, as the network structure became deeper and the quantity of convolutional layers increased, the model parameters also multiplied. Given the constraints of limited computational power and memory capacity, as well as our objective of generating high-quality images, it became crucial to strike a balance between the model’s dimensions and the quality of the images generated.

FastGAN [15] is a lightweight and efficient GAN architecture that incorporates a skip-layer channel-wise excitation module alongside a self-supervised discriminator, which is trained as a feature encoder. In this study, we chose to adopt the FastGAN generator as the fundamental architecture. The FastGAN generator utilized a single convolutional layer at each resolution and applied only three channels to the convolutional layers at higher resolutions. This minimalist design enabled the model to be more suitable for scenarios using a limited amount of data.

**Figure 8:**
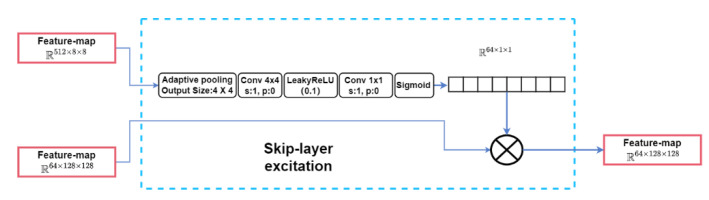
The SLE module.

To better train deep models, He et al. [21] introduced the residual block (ResBlock), which employs skip connections to reinforce the gradient flow between layers. This approach has been widely adopted in various classical GANs [22,23], although it increases the computational cost. In contrast, FastGAN introduces a skip-layer channel-wise excitation (SLE) module, which applies channel multiplication between activations to establish skip connections across a broader range of resolutions. This approach reduces the additional computational burden while maintaining a fast and efficient gradient flow. Mathematically, the SLE module is formally defined as follows:

(1) $$y=F\left(x_{low},\;\left\{W_{i}\right\}\right)\cdot x_{high}$$

where x and y denote the input feature maps and the output feature maps of the SLE module, respectively. The function F represents the operations performed on $x_{low}$, and $W_{i}$ represents the module weights to be learned. Figure 8 illustrates the SLE module connected to the 128 × 128 and 8 × 8 resolutions, where $x_{low}$ and $x_{high}$ correspond to the feature maps at 8 × 8 and 128 × 128 resolutions, respectively. Initially, F first down-sampled the feature map $x_{low}$ to 4 × 4 using an adaptive average-pooling layer. Subsequently, further down-sampling to 1 × 1 was applied using a 4 × 4 operation. After the activation function, LeakyReLU, which was a 1 × 1 convolution layer, was utilized to project $x_{low}$ into the same channel shape as $x_{high}$. Finally, after passing through a Sigmoid function, the output from F was multiplied by $x_{high}$ along the channel dimension. This multiplication yielded y, which possessed a shape identical to $x_{high}$.

**Figure 9:**
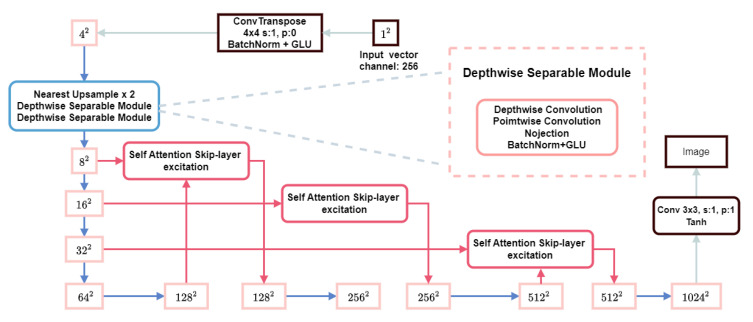
An overview of the generator.

In this study, we proposed a redesign of the generator of FastGAN by incorporating depth-wise separable convolution and the GAM (global attention module) [24] to enhance the generative capability of the network with minimal additional computational effort and without compromising the computational speed. The structure of our generator is defined as shown in Figure 9:

#### 3.3.2. Depth-Wise Separable Convolution

**Figure 10:**
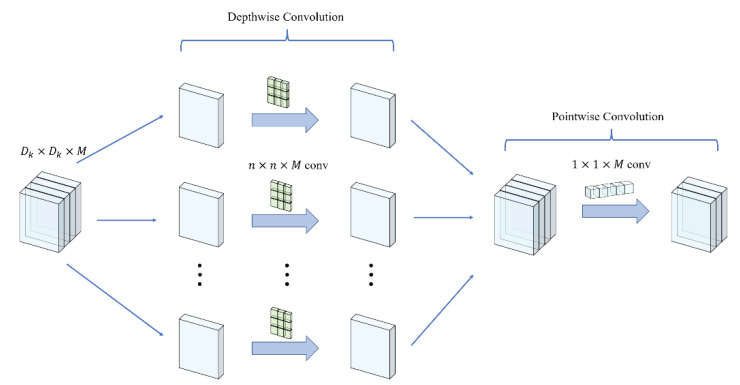
Depth-wise separable convolution.

Convolution, in the context of neural networks, is the process of extracting features from input by sliding windows over the data. It can be visualized as zooming in and capturing an image at each step using a magnifying glass and stitching these images together to form a larger picture. Convolution is currently a fundamental operation in computer vision tasks. The prevailing trend is to construct increasingly deep and complex networks to attain higher levels of accuracy. However, this often results in larger numbers of parameters and slower computation speeds. To address these challenges, depth-wise separable convolution [17] has emerged as a technique that decomposes the regular convolution into two separate parts: a depth-wise convolution and a point-wise convolution. In our network structure, the depth-wise convolution individually applied a filter to each input channel, performing a channel-by-channel convolution operation. Following this step, a point-wise convolution was applied, combining the outputs of the depth-wise convolution with a 1 × 1 convolution to achieve feature fusion between different channels. By utilizing depth-wise separable convolution, we achieved effective feature extraction while decreasing the quantity of parameters and maintaining computational efficiency. The depthwise separable convolution is shown in Figure 10:

The input feature map has the shape of $D_{k}\times D_{k}\times M$, and the scale of the convolution kernel of the depth-wise convolution is n×n×M, having a total of M kernels and grouping set to M. It was assumed that a single convolution operation was performed on each point at the spatial position of the corresponding feature map, and the total number of computations required for a single depth-wise convolution was $D_{k}\times D_{k}\times n\times n\times M$. This outcome occurred because the spatial dimension of the feature map encompassed a total of $D_{k}\times D_{k}$ points, and the amount of computation required to perform the convolution operation for each point was consistent with the scale of the convolution kernel. Since the grouping was M, the total computation of the depth-wise convolution was $D_{k}\times D_{k}\times n\times n\times M$. For the point-wise convolution, the scale of the kernel was 1×1×M, and there were N such kernels. A single point-wise convolution required a total of $D_{k}\times D_{k}\times M$ computations, meaning that the overall computation required for the point-wise convolution was $D_{k}\times D_{k}\times M\times N$. Therefore, the overall computation of the depth-wise separable convolution was $D_{k}\times D_{k}\times n\times n\times M+D_{k}\times D_{k}\times M\times N$. Using a similar analysis for the regular convolution, presuming that the scale of the input feature map was $D_{k}\times D_{k}\times M$ and the scale of the convolution kernel is $D_{F}\times D_{F}\times M,$ and there are N such convolutions, the total computation was $D_{k}\times D_{k}\times n\times n\times M\times N$. Then, the ratio of the computation of the DSC to the computation of the regular was is $\frac{1}{N}+\frac{1}{D_{F}^{2}}$. Based on the above analysis, it was clear that the efficiency of DSC was much better than that of regular convolution.

We utilized depth-wise separable convolution in place of regular convolution and refrained from incorporating an activation function or batch normalization following the depth-wise convolution. Some studies advocate for incorporating an activation function and batch normalization after depth-wise convolution to enhance the non-linear capability of a network, thereby enabling the network to better fit more complex functions. However, some recent research results suggest that omitting these components would be a better choice. Through experiments, we observed that in our architecture, using depth-wise convolution without the activation function or batch normalization led to better results.

#### 3.3.3. GASLE

Motivated by the notion that the human visual system can efficiently identify crucial regions in complex scenes, attention mechanisms were introduced into the domain of computer vision [25]. Attention mechanisms aim to emulate the internal processes of biological visual perception. When we look at an image, we do not perceive an entire image at once, instead focusing our attention on specific points of interest within the image; attention mechanisms in neural networks function in a similar manner. With limited computational resources, attention mechanisms sift through vast amounts of information to find the most relevant features, enabling the network to focus on the most important information.

In the domain of unconditional image generation, attention mechanisms have promising applications, effectively enhancing the quality and diversity of generated images. In 2015, Xu et al. [25] proposed an image-captioning model based on an attention mechanism, achieving remarkable achievements in the field of image description generation. In 2018, Woo et al. [26] introduced the attention-augmented convolutional network (AAN), which integrated an attention mechanism into the traditional convolutional network to enhance the perceptual field of the network through the self-attention mechanism, achieving significant improvement in image generation tasks. In 2019, Zhang et al. [27] used the self-attention mechanism in SAGAN to capture the long-range dependency relationship between different locations in the image, improving the performance of the generator. These studies demonstrate specific applications of attention mechanisms in image generation and highlight their potential to advance image generation tasks.

**Figure 11:**
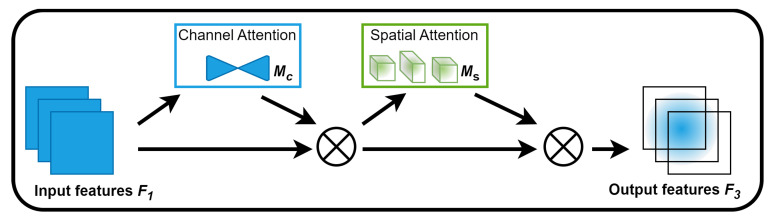
Overview of the GAM.

The self-attention mechanism has made notable advancements in the field of image generation. It improves efficiency using attention weights that consider the relationships between each pair in the three aspects of the channel dimension, spatial width dimension, and spatial height dimension, using a triplet of key, query, and value. However, the self-attention mechanism operates in two dimensions simultaneously rather than in all three dimensions. A GAM (global attention mechanism) [24] is a mechanism that reduces information loss and enhances global cross-dimensional interaction features by combining spatial and channel attention, considering the interaction between channels and spatial locations. It introduces a 3D arrangement with a multilayer perceptron. Compared to other mechanisms that solely focus on channel or spatial attention, a GAM can capture salient features in all three dimensions. In the pre-experiments included in this study, issues such as blurred edges or misrepresented colors were observed in the generated images, particularly in the trunk and disease regions. To overcome these challenges, we introduced a GAM to help the generator adjust the attention distribution across image channels and spatial locations. This action enabled increased fine-grained control over specific regions of the generated images, resulting in more realistic and detailed outputs. By leveraging the GAM, the issues of blurred edges and color errors were effectively addressed. The architecture and process of the GAM are illustrated in Figure 11, as well as Equations (2) and (3).

(2) $$F_{2}=M_{c}\left(F_{1}\right)⊗F_{1}$$

(3) $$F_{3}=M_{s}\left(F_{2}\right)⊗F_{2}$$

where $F_{1}$ denotes the input feature mapping, and $F_{2}$ and $F_{3}$ denote an intermediate state and the final output, respectively. $M_{c}$ is the channel map, and $M_{s}$ is the spatial attention map. ⊗ denotes element-wise multiplication.

**Figure 12:**
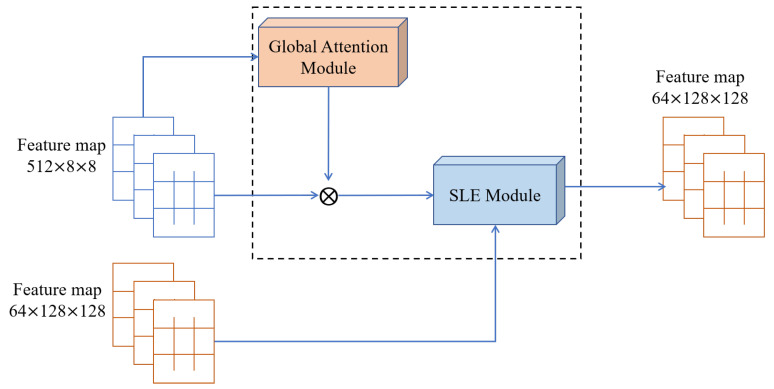
The GASLE module.

We added the GAM module to the SLE module to obtain a new module called the global attention skip-layer excitation module (GASLE). In Figure 8, FastGAN directly uses the low-resolution feature maps as inputs and passes them through the SLE module. In the GASLE module, the low-resolution feature maps were first passed through the channel attention module of the GAM to preserve the 3D information and amplify the cross-dimensional channel-space dependencies, which helped to selectively emphasize relevant channels and suppress irrelevant channels. Subsequently, in the spatial attention module, the spatial information was fused. This submodule focused on capturing spatial dependencies in feature maps, which made up for the lack of channel-only attention to some extent. Finally, the extracted and fused information from both sub-modules was integrated and underwent cross-resolution fusion with higher-level information via the skip-layer excitation module. This module helped the generator to adjust the levels of importance of different channels and reduce the loss of spatial information during the cross-resolution fusion process, as shown in Figure 12.

### 3.4. Discriminator

GANs have shown remarkable capabilities in terms of generating high-quality images. However, their successful utilization often demands meticulous regularization, extensive computational resources, and the careful fine-tuning of hyperparameters. Currently, numerous improvements for GANs have mainly been applied to generators, while relatively few improvements have been made to discriminators. In our approach, we leveraged the pre-trained multiscale discriminator ProjectedGAN as the foundational network and EfficientNet [28] as the pre-trained network to further enhance the quality, sampling efficiency, and convergence speeds of the generated images.

**Figure 13:**
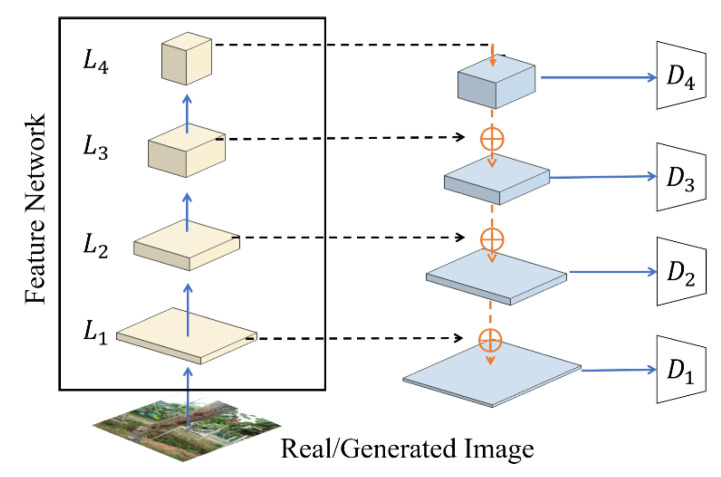
Approximate structure of the discriminator of ProjectedGAN.

Pre-training is a widely adopted technique in various computer vision tasks, offering improved training effectiveness and stability for neural networks. For the first time in the domain of unconditional image generation, ProjectedGAN introduced a pre-trained model as a discriminator and used multi-scale feedback and micro-fixable random projection to better capture deeper features. This approach ensured that the discriminator makes balanced use of all available information and prevented a heavily pre-trained discriminator from dominating the training process and causing the gradient of the generator to become extremely small. Specifically, ProjectedGAN extracted features from four layers of the pre-trained network, passed each resolution feature through a feature projector $P_{l},$ and fed it into a corresponding discriminator $D_{l}$, which employed a simple convolutional structure. The approximate structure of the framework is defined as shown in Figure 13.

### 3.5. Adamod

Training a GAN requires meticulous initialization and careful selection of the learning rate. Without appropriate hyperparameters, it will be difficult to effectively converge the loss function. During the process of training a GAN, tuning the hyperparameters often requires substantial time and patience. However, the emergence of Adam [29], which is a stochastic gradient optimization method that uses adaptive learning rates based on the square of the previous gradient, effectively reduces the workload.

Adam calculates the adaptive learning rate through utilizing first- and second-order moments. The learning rate at step t, which is denoted as $\eta_{t}$, can be computed as follows:

(4) $$\eta_{t}=\alpha_{t}/\left(\sqrt{\hat{v_{t}}}+\epsilon \right)$$

where $\alpha_{t}$ is the step size at step t, $\hat{v_{t}}$ is the bias correction of the second-order moments at step t, and ϵ is the regularization constant.

In our experiments, we observed that it became difficult to converge the network during the initial stages of training, which could be attributed to the excessive initial learning rate generated via the Adam optimizer. To address this issue, we replaced Adam with the AdaMod optimizer, which yielded a significant improvement in the convergence of the network.

AdaMod computed an exponential long-term average of the adaptive learning rate while training. It utilized this average to effectively limit excessively high learning rates without pre-heating the learning rate and with reduced sensitivity to the choice of the actual learning rate. Specifically, the operation is computed as follows:

(5) $$s_{t}=\beta_{3}s_{t-1}+\left(1-\beta_{3}\right)\eta_{t}$$

where $s_{t}$ denotes the smoothed value at the current step and acts as the interpolation between $s_{t-1}$ and the current learning rate. The hyperparameter $\beta_{3}$ is used to describe the degree of memory length in training. The range of the exponential sliding average is $1/\beta_{3}$. When $\beta_{3}$ is closer to 1, the memory length is longer. Therefore, the relationship between the smoothed value of the current step and the previously smoothed value can be calculated based on $\beta_{3}$.

(6) $$s_{t}=\left(1-\beta_{3}\right)\left[s_{t-1}+\beta_{3}s_{t-2}+\beta_{3}^{2}s_{t-3}+\cdots +\beta_{3}^{t-1}s_{0}\right]$$

Obviously, AdaMod is equivalent to Adam when $\beta_{3}$ = 0. After calculating the current smoothing value $s_{t}$, a minimum value is selected between $s_{t}$ and the current learning rate $\eta_{t}$ calculated via Adam to prune the learning rate and avoid a situation in which the learning rate is too high.

(7) $$\hat{\eta_{t}}=\min\left(\eta_{t},s_{t}\right)$$

Over the course of our experiments, we noticed that the network utilizing AdaMod converged slightly more slowly than the network utilizing Adam at the beginning of training because Adam generated an excessive initial learning rate. However, as training progressed, we noticed that it was difficult to converge the network using Adam, and it showed repeated oscillations. In contrast, the network using AdaModd maintained smooth convergence and achieved improved training outcomes.

### 3.6. YOLOv8

YOLO [30] is a single-stage object detection algorithm that mainly encompasses three key components: the backbone, neck, and head. The fundamental concept behind YOLO is to reframe the object detection task as a regression task in which the entire image serves as the input for the neural network, meaning that the network can predict the bounding box location and corresponding class of the target object.

During its development, the YOLO family has evolved. YOLOv1 was first introduced in 2016 and became a pioneer for real-time applications by processing 45 frames per second at an astonishing speed. The subsequent YOLOv2 [31] was based on the principle of Faster R-CNN and incorporated the anchor mechanism, using the K-means method to generate more representative anchor boxes, which improved the recall rate and localization accuracy. YOLOv3 [32] used Darknet-53 as the backbone, introduced the feature pyramid network to achieve multi-scale detection, and introduced logistic regression and multi-label classification to maintain a high level of detection accuracy while considering real-time constraints. YOLOv4 [33] utilized CSPDarknet53 as the backbone, employed Mosaic data augmentation to improve the detection performance of small targets, and introduced the CSPNet architecture and PANet structure to improve the ability of feature fusion and perception. YOLOv5 [34] introduced adaptive anchor frame computation and adaptive grayscale padding to solve the issue of target deformation, as well as improving the computational efficiency through the SPPF structure.

**Figure 14:**
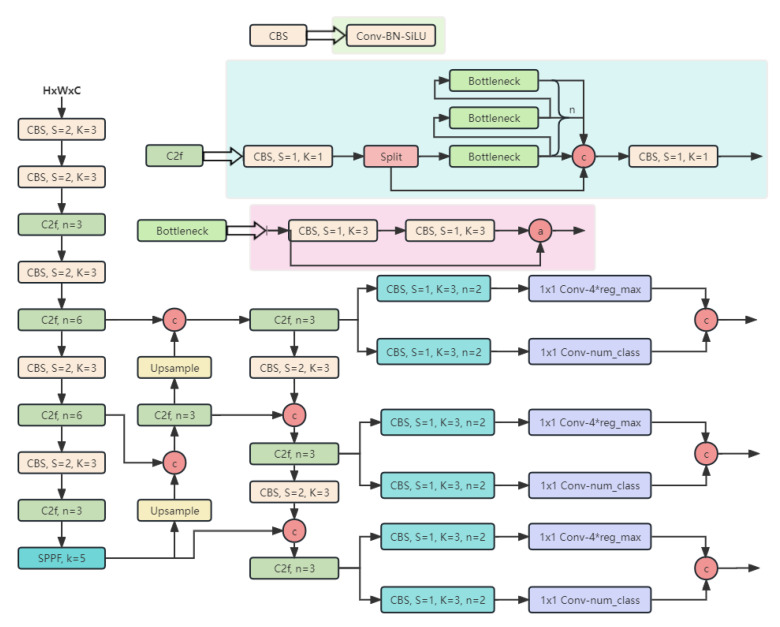
YOLOv8 structure diagram.

The YOLOv8 backbone network replaced the C3 structure of YOLOv5 with the C2f structure, which facilitated richer gradient flow and used a different number of channels to create different scales of the model, substantially enhancing the model’s performance. In terms of the head component, YOLOv8 adopted a decoupled head structure, which separated the classification and detection heads, gaining more flexibility and specialization, leading to improved performance in object recognition and localization. Furthermore, YOLOv8 replaced the anchor-based approach used in previous versions with an anchor-free method. The data augmentation part of training introduced the operation of closing Mosaic augmentation to the last 10 epochs from YOLOX [35] to effectively improve the accuracy. The structure of YOLOv8 is illustrated in Figure 14.

### 3.7. Data Augmentation Based on Image Processing Techniques

**Figure 15:**
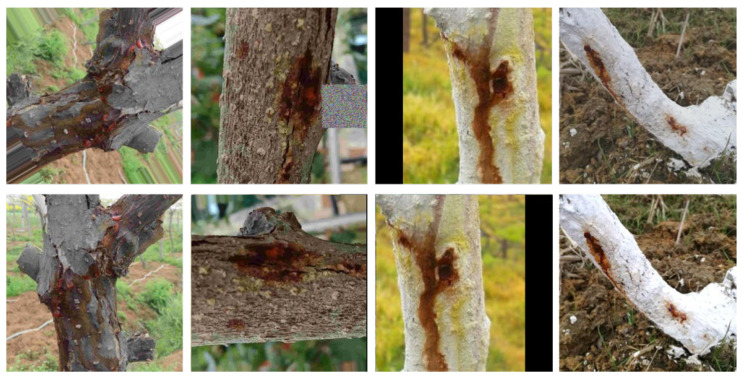
Some examples of image augmentation.

Data augmentation based on image processing techniques is a technique that artificially augments the training dataset by producing additional equivalent data from limited data through methods such as color transformation. It is an effective approach that is often used to address the scarcity of training data and extensively applied to various domains of deep learning. In our study, we applied data augmentation techniques based on image processing to the dataset. We explored various data augmentation methods and, finally, expanded the original dataset threefold, as shown in Figure 15.

During the experiments, we noticed that the detection accuracy was reduced when certain augmentation methods, such as rotation, were applied. This finding suggests that some augmentation techniques might introduce variations that are not beneficial for object detection tasks, leading to a decrease in performance. Additionally, we discovered that overfitting could become a concern if the number of data augmentations becomes excessively large.

### 3.8. Evaluation Indicators Used in This Experiment

This research adopted the Fréchet inception distance (FID) [36] as an evaluation metric to assess the capability of generative models. The FID quantifies the distances between the feature vectors of the generated images and the real images. The FID utilizes Inceptionv3 [37] network to compute the resemblance between a generated image and a real image. Inceptionv3 is a deep neural network designed to extract features, and the last layer is a pooling layer that outputs the category of an image. During FID computation, the last pooling layer was substituted with the output value of the activation function. This new output layer had a 2048-dimensional activation vector, meaning that each image was predicted as a 2048-dimensional activation feature. For the real image, this feature vector followed a normal distribution. Similarly, the 2048-dimensional feature generated via the GAN also formed a distribution. The purpose of the GAN was to minimize the dissimilarity between these two distributions and make them as similar as possible. The formula used to determine the FID is defined as follows:

(8) $$FID={\left|\left|\mu_{r}-\mu_{g}\right|\right|}^{2}+T_{r}\left(\sum_{r}+\sum_{g}-2{\left(\sum_{r}\sum_{g}\right)}^{1/2}\right)$$

where $\mu_{r}$ represents the average feature value of the real image, $\mu_{g}$ represents the average feature value of the generated image, $\sum_{r}\;$ represents the covariance matrix of the real image, $\sum_{g}\;$ represents the covariance matrix of the generated image, and $T_{r}$ represents the matrix trace.

The FID denotes the distance between the feature vectors of the generated image and the feature vectors of the real image. The shorter this distance, the better the generative model’s performance, which means that the generated image is sharp and rich in diversity.

For the object detection model, this paper used precision (*P*), recall (*R*), and the mAP as evaluation metrics. The P and R equation are presented as follows:

(9) $$P=\frac{TP}{\left(TP+FP\right)}$$

(10) $$R=\frac{TP}{\left(TP+FN\right)}$$

where a TP (true positive) denotes the accurately classified positive samples, an FP (false positive) denotes the misclassified positive samples, and an FN (false negative) denotes the misclassified negative samples.

Average precision, which is denoted as AP, is a metric that measures the area under the precision–recall (P–R) curve. The mean average precision, i.e., mAP, is the average of the mean precision of each category and indicates a comprehensive evaluation of the detection target.

## 4. Discussion

This paper presents a new pipeline to handle problems of data insufficiency in the detection of kiwifruit bacterial canker. To achieve high accuracy and real-time detection in scenarios with insufficient data, we applied an image generative model to perform data augmentation. To ensure that the images that we generated were realistic, clear, and sufficiently reliable to contribute to the detection task, we conducted an in-depth study of the generator. We introduced the GASLE module, which introduced the GAM into the generator, enabling the generator to focus on more effective information and preventing local collapse issues. In addition, we utilized depth-wise separable convolution to construct a deeper generator structure without incurring additional computational overhead. In addition, the introduction of Gaussian noise helped to enhance the diversity of the generated images. To increase training stability and accelerate convergence, we adopted the AdaMod optimizer, which effectively suppressed excessive learning rates through utilizing long- and short-term memory parameters to ensure the convergence of the network. By training the YOLOv8 model on a dataset expanded using the generated images, we achieved an accuracy of 87.7%, representing an increase of nearly 20% compared to the accuracy of the original dataset. This outcome confirms that our method is very effective. Additionally, we explored the impacts of various data augmentation methods on few-shot object detection using complex backgrounds.

In the past, research could not apply the computer vision method to rare diseases because of insufficient data. As far as we know, this approach represents a completely new strategy for the detection of plant diseases. We observe that excessive data augmentation may lead to overfitting and reduce prediction accuracy. We believe that our proposed method is suitable for most situations in which there is a lack of samples and can provide a reliable research approach for less common or rare plant diseases and plant diseases for which it is difficult to collect a substantial quantity of data. In addition, our method has perfect performance in this task, which proves that our method has achieved SOTA.

Firstly, we compared the FID of each GAN and found that our method achieved an FID of 84.18, surpassing those of all other methods. Then, we analyzed the detection effect of YOLOv8 on three datasets, and we found that YOLOv8 had the best detection effect on the database expanded using our generated images. Finally, we performed ablation experiments and found that all three improvements that we introduced into our generative model could bring positive results in terms of disease surveillance.

We believe this study has a wide range of potential applications in agriculture, horticulture, and other related fields. First and foremost, the method can help farmers and agricultural professionals to identify and respond to crop diseases in a timely manner in the face of data scarcity, thereby increasing yields and reducing losses. Secondly, in horticulture, numerous garden plants are also vulnerable to disease threats. Using this method of disease detection in garden plants, horticulturists and plant protection workers can better maintain plant health and ensure that the landscape is aesthetically pleasing and ecologically balanced. In addition, the method has the potential to be applied to environmental monitoring. Monitoring plant diseases in real time can help to detect and respond to plant health problems at an early stage, supporting environmental protection and the maintenance of ecological balance. In summary, this study has the potential for wide applications in agriculture, horticulture, and environmental monitoring, which can help to improve crop and plant health management, yields, and quality, as well as enable the sustainable development of the ecological environment.

Since this study mainly investigates kiwifruit trunk bacterial canker, its application to other plant diseases needs to be further verified. Different plant diseases may have different characteristics and manifestations and, thus, need to be optimized and adapted for specific diseases. Secondly, although this study utilizes the image generation model to achieve data expansion, the generated images may still have a certain degree of unreality. In particular, in some extreme cases, the generated images may not fully match the real-world scenes. Therefore, in practical applications, rigorous validation and calibration of the generated images are also needed to ensure that they accurately represent plant disease samples.

In the future, we hope to further combine different image generation techniques, style transfer methods, etc., to create more richly diverse samples and enhance the generalization ability of the model. In addition, we plan to construct larger and richer plant disease datasets covering different crops, environments, and disease types to extend the proposed method from single-crop disease to disease detection in more variable crops, as well as to explore its adaptability in different plant species and types. This approach will help us to build a more comprehensive smart agricultural system that provides accurate detection and prediction capabilities for a wide range of plant diseases.

## 5. Conclusions

To detect kiwifruit trunk diseases in natural scenes with insufficient data, this paper proposed a new pipeline to detect bacterial canker. In the first step, we optimized more advanced GANs to generate high-quality images. Secondly, we adopted YOLOv8 to detect the disease based on the generated images, which achieved SOTA (state of the art) status. The advanced GAN design included the integration of a GAM into the generator via the GASLE module, the utilization of depth-wise separable convolution for a deeper network structure, and the enhancement of the optimizer to effectively regulate learning rates. These improvements led to a significant decrease in the FID metric in our generative adversarial network, as well as the production of sharp, realistic, and diverse images. Using generated images to augment the original dataset, the mAP@0.5 of YOLOv8 was improved by nearly 20% compared to the detection performance based on the original dataset. Furthermore, the study investigated the impact and limitation of data augmentation in the detection of trunk bacterial canker from complex backgrounds and with insufficient data. The experimental findings reveal that when the data are augmented to four times their original size, the mAP reaches 81%, though the precision will decrease. At this point, more image augmentation methods will cause performance degradation. This study represents the first time that researchers have proposed using the GAN-related method to perform plant disease detection. After this detailed analysis, our method has achieved SOTA.

In the future, we will focus on further optimizing our generative models and implementing network-pruning techniques. Furthermore, we will continue to expand research into various types of plant diseases and explore the diverse range of plant species. These efforts will help to enhance the applicability and generalization of our methods and introduce more innovations and breakthroughs to the field of plant disease detection. Meanwhile, it will also provide referable ideas for plant disease detection and early prevention in agriculture, horticulture, or other related fields.

## Figures and Tables

**Table 1:** The FID of models.

Methods	FID
FastGAN	125.41
ProjectedGAN	86.28
Ours	84.18

**Table 2:** The results of the ablation experiments.

Methods	FID
FastGAN	125.41
ProjectedGAN	86.28
Baseline and DSC	85.71
Baseline and GASLE	86.4
Baseline and AdaMod	85.4

## Data Availability

The data presented in this study are available upon request from the corresponding author. The data used in our research are not publicly available, as they are also being utilized in an ongoing study.
